# The impact of employees’ experience of high-performance work systems on innovative behavior in professional service firms

**DOI:** 10.3389/fpsyg.2023.1324474

**Published:** 2024-01-08

**Authors:** Beenish Arshad, Hamid Hassan, Akbar Azam

**Affiliations:** FAST School of Management, National University of Computer and Emerging Sciences, Lahore, Pakistan

**Keywords:** high performance work system, innovative work behavior, knowledge sharing, need for cognition, professional service firms, social capital

## Abstract

This research examines the impact of employees’ experience of high-performance work systems (HPWS) on their innovative behavior. The study draws upon social exchange theory to propose that employees’ experience of HPWS influences their innovative behavior directly and through sequential mediation of social capital development and knowledge-sharing behavior. Additionally, the study uses the Elaboration Likelihood Model to highlight that individuals’ need for cognition strengthens the relationship between employees’ knowledge-sharing and innovative behaviors. The study employed a time-lagged quantitative research design with survey data from 262 job incumbents in professional service firms. The proposed model was tested using the PLS-SEM two-stage approach. The findings of the study confirm the proposed direct and indirect relationships. Moreover, the findings also confirm that the need for cognition strengthens the relationship between knowledge-sharing and individual innovation behaviors. The study posits that employees’ experience of HRM systems can influence their innovative behavior as a reciprocal exchange toward the employer. Moreover, this study presents a comprehensive model that highlights the interplay of social and cognitive factors that can influence the relationship between HPWS and employees’ innovation behavior. This study also fills a gap in the existing literature by highlighting the antecedents of innovative behavior in professional service firms.

## 1 Introduction

The Social Exchange Theory (SET) is a prominent conceptual paradigm for understanding employees’ behaviors in workplaces. SET is based on the premise that individuals develop and maintain those social relationships in which benefits outweigh the costs (Blau, 1964; Cropanzano and Mitchell, 2005). Social relationships are sustained when individuals abide by the “norm of reciprocity” (Gouldner, 1960). The norm of reciprocity refers to socially accepted rules for social transactions in which one party receives a benefit and is mutually obligated to return the benefit to the other party. Also, it facilitates relationship maintenance, cooperation, and exchange behaviors between individuals (Belmi and Pfeffer, 2014).

In organizational contexts, the SET and its tenets can be used to understand norms that shape interpersonal relationships and reciprocal obligations reflected in employee behaviors (Oparaocha, 2016). In this paper, we have used the SET to investigate how employees’ experience of high-performance work systems influences innovative behavior (IB) in professional service firms (PSFs). Employees’ IB refers to the intentional generation, promotion, and application of novel and useful ideas in a work role, group, or organization (Janssen, 2000). Existing research has highlighted that factors such as personality traits, job design, organizational climate, and leadership styles promote innovation in organizations (Shanker et al., 2017; Woods et al., 2018; Mustafa et al., 2022; Hoang et al., 2023; Tsamantouridis et al., 2023). Despite the growing academic interest in the antecedants of innovation, there is still dearth of knowledge regarding the factors and mechanisms that drive individual level innovation particularly in PSF contexts. PSFs are knowledge-intensive firms that rely on specialized human resources to deliver customized and unique service offerings to clients. Therefore, employees’ IB contributes toward improving work processes and developing innovative service offerings (Fu et al., 2015).

A high-performance work system (HPWS) is an integrated set of human resource practices that are designed to enhance employee motivation, competence, commitment, and performance (Aryee et al., 2012). HPWS inculcates practices such as selective staffing, extensive training and development, work autonomy, result-oriented reward systems, participative work designs, and employment security (García-Chas et al., 2014). Considering the tenets of SET, our first argument is that employees demonstrate IB as a reciprocal behavior in exchange for inducements received from the HPWS. Prior research indicates that HPWS shapes positive employee attitudes and behaviors through participation, autonomy, and motivation (Kehoe and Collins, 2017). Employees perceive HPWS as an organization’s investment and its commitment to establishing long-term relationships with employees (Takeuchi et al., 2007; Alfes et al., 2021). Due to these perceptions, social exchange relationships evolve between employees and the organization (Zhang et al., 2019). In a social exchange relationship, employees demonstrate commitment and discretionary behaviors in exchange for benefits received from the employer (Zhang et al., 2019; Asante et al., 2023).

The objective of HPWS is improve organizational performance through a collaborative work environment, active employee participation, and involvement (Patel et al., 2013; Wang et al., 2023). When HPWS is implemented, employees perceive a mutual obligation to fulfill its objective by demonstrating commitment and social integration (Evans and Davis, 2005; Donate et al., 2020). Consequently, relationship building, cooperation and resource exchanges can result in exchange for benefits received from HPWS (Jiang and Liu, 2015; Zhang et al., 2019). We consider these arguments to propose that HPWS can influence the development of internal social capital in organizations. According to Singh et al. (2021), social capital facilitates knowledge sharing, a form of resource exchange between coworkers. Furthermore, knowledge sharing facilitates value creation through innovative idea generation and application in organizations (Radaelli et al., 2014; Pian et al., 2019). Hence, in line with these arguments, we also propose a sequential mediation model that reflects that employees’ experience of HPWS facilitates IB directly and through social capital (SC) development and knowledge-sharing (KS) behaviors. Thus, this paper contributes to the debate that human resource practices create supportive social architecture that facilitates resource exchange and innovation (Oparaocha, 2016).

Prior research has shown that KS drives employees’ IB (Pian et al., 2019). However, due to cognitive differences, not all individuals may demonstrate similar levels of IB. In this study, we investigate whether the relationship between KS and IB is contingent on the level of an individual’s need for cognition (NFC). NFC refers to an individual’s dispositional tendency to engage in analytic information processing, deep thinking, and cognitive elaboration (Cacioppo and Petty, 1982). The Elaboration Likelihood Model (ELM) (Petty and Cacioppo, 1986) is used to explain that an individual’s NFC influences the range and depth of information processing. According to Wu et al. (2014), NFC aligns with the cognitive demands for the generation, evaluation, and implementation of innovative ideas. In this study, we suggest that NFC can influence IB through in-depth and analytical processing of knowledge shared during social interactions. Therefore, NFC can be considered a relevant cognitive boundary condition while studying the relationship between KS and IB. By examining the moderating role of NFC on the relationship between KS and IB, we intend to enhance the understanding of the cognitive factors that play a role in promoting IB in the workplace.

The study seeks to answer that to what extent and through which mechanisms do employees’ experience of HPWS impact their innovative behavior in PSFs. Furthermore, this study addresses how individuals’ need for cognition influences the relationship between knowledge sharing and innovative behavior. The motivation for this research stems from the need to understand “how” interrelated HR practices influence innovation in PSFs. The study adopts an in-depth approach and highlights the combined interplay of social and cognitive factors that link HPWS and IB. Moreover, existing studies have predominantly focused on understanding the outcomes of HPWS, using a managerial perspective. Recent studies suggest that employees have varied perceptions about human resource systems (Ali et al., 2022; Kim et al., 2023). Thus, this study takes into account employees’ perspectives of human resource systems and the resultant impact on their behaviors. Furthermore, existing studies on HR practices and innovation have been widely conducted in manufacturing and high-technology companies in developed country contexts (Park et al., 2017; Bos-Nehles and Veenendaal, 2019; Yao et al., 2023). Thus, to fill the research gap, this study uses empirical data from employees in PSFs in Pakistan, an emerging Asian country. Furthermore, there is less research on the role of cognitive factors in influencing individual innovation behaviors in workplaces. Thus, this study highlights the role of NFC, a boundary condition that can strengthen the relationship between KS and IB. The findings of this study offer valuable practical understanding to managers who can leverage the benefits of HR practices and resultant employee behaviors for PSFs’ competitive advantage.

## 2 Theoretical background and hypotheses

### 2.1 Employee experience of HPWS and innovative behavior

A high-performance work system (HPWS) is a set of internally coherent human resource practices designed to enhance employees’ commitment and performance (Gong et al., 2010; Van De Voorde and Beijer, 2015). The social exchange theory (SET) is often used to explain the impact of specific human resource practices on employees’ behaviors (Bos-Nehles et al., 2017). Recently, Kim et al. (2023) used the SET to assert that HPWS increases employees’ IB through perceived organizational support and perceived leader support as a boundary condition. Prior studies suggest that employees view HPWS as an organizational investment in their development and well-being. As a result, they reciprocate by demonstrating positive attitudes and investing their time to look for ways to improve work performance (Zhang et al., 2019; Pahos and Galanaki, 2022). HRM practices, such as employee training programs are perceived as an organization’s personalized commitment toward employees’ development and growth. When employees receive developmental opportunities, they demonstrate positive behaviors and attitudes which may or may not be part of job descriptions (Bos-Nehles et al., 2017).

Janssen (2000) argued that employees’ perceptions of fair reward systems instill a reciprocal obligation to demonstrate IBs. Ramamoorthy et al. (2005), further extended this idea and explained that the relationship between specific HR practices (e.g., job autonomy and fair pay) and IB is mediated through perceptions of psychological contract fulfillment. Moreover, according to Zhu et al. (2022), employees develop and implement innovative ideas in the workplace in exchange for reasonable work autonomy and rewards. Employee support and employment security enhance employees’ confidence to take initiative and communicate their ideas to managers (Bos-Nehles et al., 2017). Based on the these arguments, we propose the following hypothesis:

*H1*: Employees’ experience of HPWS is positively related to their innovative behavior.

### 2.2 Employee experience of HPWS and social capital

SC refers to the resources available through, embedded within, and derived from networks of relationships. SC is represented by three components: the strength of interpersonal relationships, interpersonal trust, and shared vision (Nahapiet and Ghoshal, 1998). According to Evans and Davis (2005), interrelated HRM practices create an enabling social context for relationship building and collaboration. Evans and Davis (2005) identified organizational social structure as a mediating variable between HPWS and organizational performance. They further highlighted specific practices such as selective staffing, training programs, and internal mobility facilitate social integration and the formation of bridging social ties between coworkers. According to Patel et al. (2013), elements of an HR system help build a workplace context characterized by coworker support and trust.

HPWS inculcates flexible and team-based work designs which increase the frequency of interactions between coworkers (Wang et al., 2023). Moreover, performance-based reward systems enhance employees’ commitment to achieving the collective goals of the organization (Patel et al., 2013; Wang et al., 2023). Recently, Donate et al. (2020) studied a sample of TQM-based Spanish organizations. They assert that the TQM philosophy is linked to HPWS adoption and subsequently SC development in organizations. HPWS adoption leads to the development of social relationships, interpersonal trust, and shared vision among coworkers (Jiang and Liu, 2015; Donate et al., 2020). Given these arguments, we propose the following hypothesis:

*H2*: Employees’ experience of HPWS is positively related to the development of social capital.

### 2.3 Social capital and knowledge sharing

SC provides individuals with access to knowledge and expertise embedded within social relationships (Ganguly et al., 2019). According to Inkpen and Tsang (2005), SC serves as a bonding element that promotes intra-network KS behaviors. Moreover, it facilitates knowledge search activities within social networks (Alguezaui and Filieri, 2010).

Interpersonal trust, an integral component of SC prevents opportunistic behaviors and increases resource exchange between coworkers (Nahapiet and Ghoshal, 1998; Inkpen and Tsang, 2005). According to Santos et al. (2023), interpersonal trust increases an individual’s inclination toward sharing knowledge with peers, while a lack of trust serves as a barrier to KS. Yen et al. (2015), argue that interpersonal trust and closely knit social relationships influence individuals’ intentions to voluntarily contribute their knowledge to coworkers.

Lefebvre et al. (2016) argued that SC plays an important role in increasing KS performance in learning networks. Studies suggest that features of social networks have an impact on knowledge transfer in organizations. For example, relationship strength increases tacit KS and integration (Reagans and McEvily, 2003; Ganguly et al., 2019). Reagans and McEvily (2003) emphasized that cohesiveness within relationships affects motivation and willingness to invest time, energy, and effort toward KS. According to Singh et al. (2021), cohesive social relationships facilitate knowledge flow within organizations. Moreover, cohesive social relationships increase the frequency of KS interactions among coworkers (Al-Omoush et al., 2022). In view of these arguments, we propose the following hypothesis:

*H3*: Social capital is positively related to employee knowledge-sharing behavior.

### 2.4 The mediating role of social capital between employees’ experience of HPWS and knowledge sharing

We propose that SC mediates the relationship between HPWS and KS. Although there is some evidence that HPWS facilitates social interactions and cooperation among coworkers, the link between HPWS and KS through SC remains underexplored in the existing literature. Prior studies indicate that HPWS facilitates the development of social exchange relationship between employee and the employer (Zhang et al., 2019). In a social exchange relationship, employees demonstrate shared behavioral and attitudinal responses conducive to the collective achievement of organizational goals (Gong et al., 2010). In this context, they are likely to demonstrate cooperation and coordination which drives value creation (Ganguly et al., 2019; Donate et al., 2020).

Scholars posit that HPWS facilitates the development of a social climate that encourages resource sharing (Bartram et al., 2021). According to Wang et al. (2023), when HPWS inculcates team-based reward systems, employees’ are motivated to cooperate and share their resources to achieve collective goals. According to Swart and Kinnie (2003), human resource practices and policies act as facilitators of SC formation. Kehoe and Collins (2017) argued that integrated human resource systems contribute to unit performance by fostering interpersonal exchange conditions and facilitating knowledge flow within the organization. Furthermore, Singh et al. (2021) argued that knowledge-based human resource practices promote KS interactions through relationship building and networking among coworkers. HPWS enhances relational coordination, thereby allowing employees to combine and exchange knowledge (Collins and Smith, 2006; Siddique et al., 2019). Given these arguments, we propose the following hypothesis:

*H4*: Social capital mediates the relationship between employees’ experience of HPWS and their knowledge-sharing behavior.

### 2.5 Knowledge sharing and innovative behavior

Existing literature highlights that knowledge is a strategic resource and effective utilization of knowledge is important to drive innovation in organizations (Subramaniam and Youndt, 2005; Kmieciak, 2020). KS is a social exchange behavior that involves the collection and donation of knowledge between individuals (Van Den Hooff and Ridder, 2004).

Munir and Beh (2019) asserted that KS allows flow and integration of knowledge, which prompts new idea generation and application. KS between coworkers facilitates task accomplishment and identification of opportunities for improving products, services, and work processes. According to Pian et al. (2019), KS interactions facilitate new knowledge creation and integration which allows the generation of novel ideas and solutions at work. The process of KS drives cognitive structuring by allowing individuals to connect existing knowledge with new knowledge. Tsai and Zheng (2021) studied the mediating role of KS between employee curiosity and service creativity. They highlighted that service creativity is enhanced through collection and donation of knowledge between coworkers. According to Castaneda and Cuellar (2020), KS increases the innovativeness of sharers in terms of capacity and propensity to promote and implement new ideas. During KS interactions, individuals reflect and elaborate on diverse perspectives, thereby driving creative idea generation and application (Radaelli et al., 2014). Given these arguments, we propose the following hypothesis:

*H5*: Employee knowledge-sharing behavior is positively related to their innovative behavior.

### 2.6 The mediating role of knowledge sharing between social capital and innovative behavior

Existing literature supports the notion that cohesive relationships facilitate KS and different forms of innovation in varied organizational contexts (Ganguly et al., 2019; Singh et al., 2021). However, the precise intermediary role of KS between SC and individual-level innovation needs further exploration. We argue that KS mediates the relationship between SC and IB. According to Ganguly et al. (2019), strength of social relationships and interpersonal trust drive KS and firm innovation performance.

Subramaniam and Youndt (2005) posited that innovation is a collaborative effort that is driven by the exchange of knowledge within an organization. SC drives innovation through collaborative social interactions between coworkers (Donate et al., 2020). Existing studies have used heterogeneous samples from different industries to argue that cohesive relationship ties facilitate knowledge creation and the development of employees’ innovative capabilities (Akhavan and Mahdi Hosseini, 2015; Ganguly et al., 2019). Empirical evidence suggests that SC facilitates radical and incremental innovation by allowing employees to search for and share knowledge within social networks (Alguezaui and Filieri, 2010).

The process of innovation requires an enabling social context characterized by sharing and utilization of knowledge held by individuals (Yang et al., 2018). According to Al-Omoush et al. (2022), innovation is driven by strong social ties and transformation of tacit knowledge between network participants. According to Akram et al. (2020), IB depends on social support, cooperation, and knowledge acquired through social interactions. Thus, given these arguments, it can be inferred that KS acts as a mediating mechanism that influences the relationship between SC and IB. Thus, we propose the following hypothesis:

*H6*: Employees’ knowledge-sharing behavior mediates the relationship between social capital development and employees’ innovative behavior.

The above arguments suggest a sequential mediation of SC and KS between employee experienced HPWS and IB. Prior studies have emphasized that integrated HR practices promote relationship building, collaboration, and resource sharing (Evans and Davis, 2005; Donate et al., 2020). HPWS acts as a catalyst for SC development in organizations. As suggested by prior studies, SC can promote employees’ innovation behavior by providing an access to expertise and knowledge embedded within networks of relationships (Ganguly et al., 2019; Al-Omoush et al., 2022). Employees’ experience of HPWS promote cooperative social interactions, interpersonal trust, and shared vision which drive employees’ knowledge sharing and innovation behaviors in organizations (Jiang et al., 2012; Akram et al., 2020; Al-Omoush et al., 2022). In line with these arguments, we suggest a sequential link between employee experienced HPWS and IB. Thus, we propose the following hypothesis:

*H7*: Social capital and knowledge-sharing behavior sequentially mediate the relationship between employees’ experience of high-performance work systems and their innovative behavior.

### 2.7 The moderating role of employees’ need for cognition

Need for cognition (NFC) is an individual’s dispositional tendency to engage in deep thinking and analytical information processing (Cacioppo and Petty, 1982). The Elaboration Likelihood Model (ELM) (Petty and Cacioppo, 1986) can be used as a theoretical basis to explain the moderating role of NFC in influencing the relationship between KS and IB. The ELM is a dual-process model that explains how individuals process information and make decisions about the amount and nature of the mental processing they engage in. According to ELM, there are two routes of information processing: central and peripheral. The central route involves in-depth information processing, critical thinking, and deep cognitive elaboration. The peripheral route processing involves surface thinking and reliance on heuristics for decision-making (Smith and DeCoster, 2000).

Individuals with high NFC demonstrate a tendency toward central route processing. They thoroughly analyze information and consider diverse perspectives in decision-making. High NFC allows individuals to engage in and enjoy novel, complex, and uncertain situations (Cacioppo and Petty, 1982). They seek and draw information from their environment to solve complex problems. According to Pan et al. (2021), high NFC allows individuals to capitalize on the flow of knowledge to address work-related situations. These individuals connect and utilize new and existing knowledge to generate and apply creative ideas. Individuals with high NFC seek intellectually stimulating interactions in their environment. KS interactions allow individuals with high NFC to engage in intellectual debates. Intellectual stimulation leads to creative thinking and exploration of new ideas and approaches. During social interactions, individuals with high NFC demonstrate persuasiveness by presenting thoughtful arguments to support their ideas and insights (Wu et al., 2014). Although Wu et al. (2014) established a direct link between NFC and IB, this study examines NFC as a boundary condition that helps process new and existing knowledge. NFC helps in linking new and existing knowledge acquired in social interactions, which drives creativity (Evans et al., 2003). Thus, it can be argued that individuals with high NFC are expected to exhibit proactive approaches toward IB by utilizing existing and new knowledge acquired through their interactions with coworkers. Hence, we propose the following hypothesis:

*H8*: The need for cognition strengthens the relationship between employee knowledge-sharing and innovative behavior.

**Figure 1:**
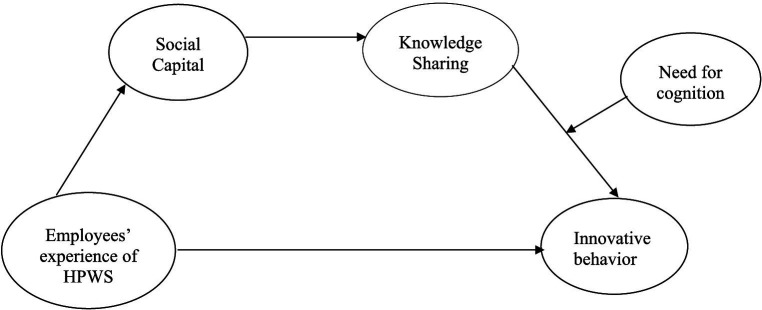
Conceptual framework.

Figure 1 shows the proposed conceptual framework of this study.

## 3 Materials and methods

### 3.1 Sample and procedure

This study employed quantitative research methodology to test the proposed model. Using a structured questionnaire, data were collected from a sample of PSFs’ job incumbents in Pakistan’s information technology, marketing, architecture, and engineering sectors. PSFs attract, retain, develop, and maintain skilled talent which delivers customized service offerings to clients. The firms chosen for this study are knowledge intensive and rely on specialized human resources to gain competitive advantage.

Initially, the authors created a list of PSFs for initial screening and preliminary communication before data collection. The chosen organizations were characterized by flat organizational structures, team-based work designs, and open communication between managers and employees. The authors used personal and professional networks to contact the managers of the listed companies. A few managers did not respond or refused to cooperate in the data collection process. Those managers who agreed to cooperate were approached for semi-structured screening interviews. The interviews included questions to assess whether the identified organizations implemented common practices of HPWS. The time duration of each interview was approximately 30 min. The study participants were chosen through convenience based sampling technique from the chosen PSFs. Covenience sampling technique helps researchers to gather data in an accessible and cost effective manner (Speak et al., 2018; Javed and Khan, 2023).

Following the initial assessment, the authors acquired permission to proceed with data collection from specialist PSF employees at different organizational levels. To increase the response rate, the authors built rapport with managers and employees to gain their support and increase cooperation. The first page of the questionnaire included a confidentiality statement which assured that all the responses would remain confidential and would be used for academic research purposes only. The questionnaire also included a statement through which participants were assured that their participation was voluntary and that they could withdraw from the study at any point in time. Furthermore, the authors ensured that the questionnaire items were concise, clear, and easy to understand.

To minimize common method variance (CMV) and create proximal separation, data were collected at three-time points 3 weeks apart. At Time 1, data on demographic characteristics (gender, age, tenure, and education) and employees’ experience of high-performance work systems were collected. At Time 2, data regarding employees’ perceptions of social capital, knowledge sharing, and employees’ need for cognition were collected. At Time 3, data on employees’ innovative behavior was collected. Each participant was assigned a unique identifier code that remained consistent across all time points. The identifier ensured that responses could be matched across different data collection time points. One to one matching of responses was carried out at the end of data collection process.

We intended to use PLS-SEM for the purpose of data analysis. PLS-SEM is used to analyze complex models and facilitates data analysis with small sample sizes (Hair et al., 2019). We reviewed recommendations from Kock and Hadaya (2018) to estimate the minimum sample size. According to Kock and Hadaya (2018), the minimum sample size for PLS-SEM is recommended to be 160 when magnitudes of path coefficients cannot be reasonably estimated. The value of the minimum sample size is based on the inverse square root method. The sample size of this study was above the minimum recommended value based on the inverse square root method. A total of 375 questionnaires were administered to the potential respondents in 45 companies. The number of administered questionnaires was significantly higher than the minimum required sample, considering response rate considerations. The response rate was approximately 70%, and 262 matched responses were retained for the analysis. Out of 262 responses, 85 responses were obtained from IT companies, 63 responses were obtained from marketing companies and 114 responses were obtained from engineering and architecture companies. The demographic profile of the respondents is shown in Table 1.

**Table 1:** Demographic profile of respondents.

Demographic		Frequency	Percentage (%)
Gender	Male	144	55
	Female	118	45
Age	Less than 25	52	19.8
	25–34 years	136	51.9
	35–44 years	39	14.9
	45–54 years	20	7.6
	55 above	15	5.7
Qualification	Undergraduate	113	43.1
	Graduate	128	48.9
	Doctorate	6	2.2
	Other	15	5.7
Hierarchical Level	Entry	88	33.6
	Middle	139	53
	Senior	35	13.3
Tenure	Less than 5 years	99	37.8
	5 to 10 years	70	26.7
	11 to 15 years	31	11.8
	More than 15 years	62	23.6

*N* = 262.

### 3.2 Measurement scales

The measurement scales used in this study are described below. All items were measured using a 5-point Likert Scale. Some items were reworded to increase clarity and reduce ambiguity while maintaining the original meaning. The questionnaire items are provided in Appendix 1.

#### 3.2.1 Employee experience of HPWS

A 12-item scale adapted from Edgar et al. (2021) was used to measure employee experience of HPWS. The instruments measured the extent to which employees agreed that they experienced practices, such as selective staffing, training and development, decision-making empowerment, participation, team based work designs, and compensation contingent on performance.

#### 3.2.2 Social capital

A 12-item scale adapted and modified from Leana and Pil (2006) and Tsai et al. (2014) was used to measure employee perceptions of SC. Tantardini and Kroll (2015) recommended that SC should be measured using multiple informant surveys to capture individual perceptions of social relationships, interpersonal trust and shared vision. Furthermore, based on the recommendations of previous studies (Leana and Pil, 2006; Parzefall and Kuppelwieser, 2012), SC was measured as a unidimensional construct by averaging item scores.

#### 3.2.3 Knowledge sharing

Employees’ KS behavior was measured using a 7-item scale adapted from Lin (2007). The scale comprises items that measure the extent to which employees collect and donate knowledge from their coworkers.

#### 3.2.4 Innovative behavior

Employee IB was measured using Janssen’s (2000), 9-item scale. The scale measured respondents’ agreement regarding their idea generation, promotion, and realization behaviors in the workplace.

#### 3.2.5 Need for cognition

This study used the 6-item short Need for Cognition Scale (NFC-6) developed by Lins de Holanda Coelho et al. (2020). The scale is a shortened version of the original NFC-18 (Cacioppo et al., 1984). Lins de Holanda Coelho et al. (2020) demonstrated that the NFC-6 is a reliable, parsimonious, and valid measure of individuals’ need for cognition.

### 3.3 Assessment of common method variance

Self-reports were used to measure the key study variables. Therefore, as recommended by Podsakoff et al. (2012), we used procedural and statistical techniques to assess and control for common method variance (CMV). The procedural remedy included ensuring anonymity and confidentiality of the responses so that biased responses could be mitigated. Moreover, we added additional variables to the questionnaire to create psychological separation and prevent respondents from inferring relationships between the constructs.

The statistical methods included the application of two statistical tests to assess the CMV. First, we conducted Harman’s single-factor test. The results of this test indicated that the total variance explained by one factor was below the threshold limit of 50%, indicating that CMV was not significant. Moreover, we used a collinearity test to check variance inflation factors (VIF). According to Kock (2012), CMV is a concern when the VIF values are above 3.3. In our results, VIF values ranged from 1.35 to 2.50, which confirms that CMV was not a significant concern in this study.

## 4 Data analysis and results

Partial Least Squares Structural Equation Modeling (PLS-SEM) was employed to examine the proposed relationships. PLS-SEM is a variance-based structural equation modeling technique that employs a combination of indicator variables as proxies for conceptual variables (Hair et al., 2012). PLS-SEM is widely used in the social science discipline because it does not require stringent normality assumptions and can be used to analyze complex models. Anderson and Gerbing (1988) recommended a two-stage approach to analyze the measurement (outer) and structural (inner) models. The SmartPLS 4.0.9.0 software was used as the analysis tool.

### 4.1 Assessment of measurement model

To assess the measurement model quality, the indicator loadings, internal consistency, construct reliability, and convergent validity of the items were assessed. Item loadings demonstrate how well an item represents an underlying construct. Although the recommended value of item loadings is above 0.70 (Vinzi et al., 2010), social science researchers frequently obtain loadings below this threshold. Thus, item loadings above 0.6 were retained. Lower-loading items were deleted when there was a significant improvement in Average Variance Explained (AVE). Moreover, Cronbach’s alpha (CA) and Composite reliability (CR) were assessed. The scores exceeded the minimum threshold of 0.6, indicating acceptable construct reliability and internal consistency of the items (Hair et al., 2017).

The convergent validity of the latent constructs was established by checking the Average Variance Explained (AVE) values. The results indicate that the AVE values were close to the recommended threshold of 0.5, indicating reasonable convergent validity (Hair et al., 2012). According to Fornell and Larcker (1981), the AVE is a conservative estimate of model validity. Composite reliability alone can indicate that the construct is adequate even when AVE is below 0.50. The composite reliability of all the constructs was above the recommended level; thus, the internal reliability of the measurement items is acceptable.

The results of the measurement model assessment are presented in Table 2.

**Table 2:** Assessment of measurement model.

Items	Item loadings	CA	CR	AVE
**Employee experience of HPWS**		0.88	0.90	0.48
At work I have opportunity to participate in decision-making	0.75			
I have a great deal of autonomy in the way I carry out my job.	0.65			
I have good job security.	0.74			
My organization gives fair performance-based rewards.	0.71			
Training and development opportunities are provided.	0.69			
My workplace sets very high standards and is very selective when recruiting staff.	0.67			
Managers provide developmental feedback to improve performance.	0.68			
I am provided with opportunities to express my ideas about how processes can be improved in this organization.	0.70			
I believe my values fit well with those of my organization.	0.69			
My organization values my ability to work well within a team environment.	0.65			
**Social capital** In my organization:		0.90	0.91	0.51
Employees demonstrate strong cohesiveness in workgroups.	0.63			
Employees have frequent contact with coworkers.	0.72			
Employees spend a lot of time interacting with each other.	0.68			
Employees have close social relationships with each other.	0.73			
There is a commonality of purpose among the employees.	0.69			
Employees enthusiastically pursue collective goals and mission.	0.76			
Employees are committed to the goals of the team/department/work unit.	0.68			
Employees share similar ambitions and vision.	0.72			
We can rely on co-workers and superiors, with whom we work.	0.73			
Employees have confidence in one another.	0.77			
**Knowledge sharing**		0.81	0.85	0.49
When I have learned something new, I share my knowledge with my colleagues.	0.60			
I share important work-related information with my colleagues.	0.70			
I regularly tell my colleagues about my skills and tasks performed at work.	0.72			
When I need certain knowledge, I ask my colleagues about it.	0.66			
I like to be informed of what my colleagues know.	0.79			
When I need to learn something, I ask my colleagues who have their technical knowhow, skills and abilities.	0.71			
**Need for cognition**		0.89	0.90	0.69
I would prefer complex rather than simple problems.	0.83			
I like to handle situations that require a lot of thinking.	0.85			
I find satisfaction in finding solutions to problems by thinking about them for a long time.	0.83			
I prefer my life to be filled with interesting puzzles that I must solve.	0.86			
I would prefer a task that is intellectual, challenging, and thoughtful to one that does not require much thought.	0.79			
**Innovative behavior**		0.85	0.88	0.53
I often generate ideas to tackle complex issues at work.	0.78			
I search out new working methods, techniques, or instruments for work.	0.75			
I generate original and effective solutions for challenges faced by my team/work unit.	0.70			
Where possible, I tend to mobilize support for innovative ideas given by my colleagues.	0.72			
When I have an innovative idea, I talk to my manager(s) for approval.	0.70			
I often encourage my colleagues to discuss new and innovative ideas for product/service/process improvements.	0.71			
I adopt systematic ways of introducing innovative ideas into the work environment.	0.72			

CA, Cronbach Alpha; CR, Composite Reliability; AVE, Average Variance Extracted. The table excludes the items that were dropped due to low loading values.

The discriminant validity of the model was assessed using Fornell and Larcker’s (1981) criteria. Discriminant validity is established when the square root of the AVE of each construct is higher than its correlation with other latent constructs (Fornell and Larcker, 1981). As shown in Table 3, the square root of the AVE value for each construct was higher than its correlation with the other latent constructs. The correlations between the study variables and the demographic variables are shown in Table 3. The correlations between study variables were significant at *p* < 0.01 level. The correlations between the study constructs and demographic variables were small and insignificant. The overall results confirmed that the measurement model was adequate for the structural analysis (Hair et al., 2017).

**Table 3:** Discriminant validity estimates and correlations of study variables.

	(1)	(2)	(3)	(4)	(5)	(6)	(7)	(8)	(9)
HPWS (1)	**0.69**								
IB (2)	0.26**	**0.72**							
KS (3)	0.24**	0.39**	**0.70**						
NFC (4)	0.23**	0.38**	0.44**	**0.83**					
SC (5)	0.38**	0.26**	0.34**	0.25**	**0.70**				
Gender (6)	−0.09	0.05	0.06	0.09	0.05	1			
Age (7)	−0.03	0.02	0.07	0.04	0.06	0.011	1		
Education (8)	−0.02	−0.06	0.02	0.02	0.08	0.027	0.264**	1	
Tenure (9)	−0.09	−0.03	0.06	0.05	0.07	0.044	0.753**	0.253**	1
Mean	3.61	3.83	4.14	4.15	3.74				
Standard deviation	0.69	0.60	0.54	0.65	0.56				

Bold and Italic elements in parenthesis are square root of AVE. **Correlation is significant at 0.01 level (2 tailed).

### 4.2 Assessment of structural model

For the structural model assessment, we followed the recommendations of Hair et al. (2017) and Ringle et al. (2020). As previously discussed, the VIF values indicated no potential collinearity, which could bias the path coefficients. Next, we checked the coefficient of determination (*R*^2^) to determine predictive accuracy. The proposed model accounted for a 24% variance in IB (*R*^2^ = 0.24). Furthermore, we checked the predictive accuracy and cross-validated the redundancy index (*Q*^2^). All *Q*^2^ values were above zero, indicating the predictive accuracy of the model (Hair et al., 2017).

The path coefficients and respective significance values were assessed. Hypotheses testing was conducted using a bootstrapping procedure with 5,000 sub-samples and 95% bias-corrected confidence intervals. Initially, the proposed direct relationships were analyzed. Hypothesis 1 predicted a direct and positive relationship between employees’ experience of HPWS and IB. The results demonstrated a significant direct and positive relationship (β = 0.14, *p* < 0.05). Hypothesis 2 predicted a direct and positive relationship between employees’ experience of HPWS and SC development. The results indicated that the relationship was positive and significant (β = 0.38, *p* < 0.01). Hypothesis 3 predicts a direct and positive relationship between SC and KS. Path coefficients are positive and significant (β = 0.34, *p* < 0.01), respectively. Furthermore, as hypothesized by Hypothesis 5, we assessed a positive and direct relationship between KS and IB. The results indicated a significant direct and positive relationship between KS and IB (β = 0.26, *p* < 0.01).

**Figure 2:**
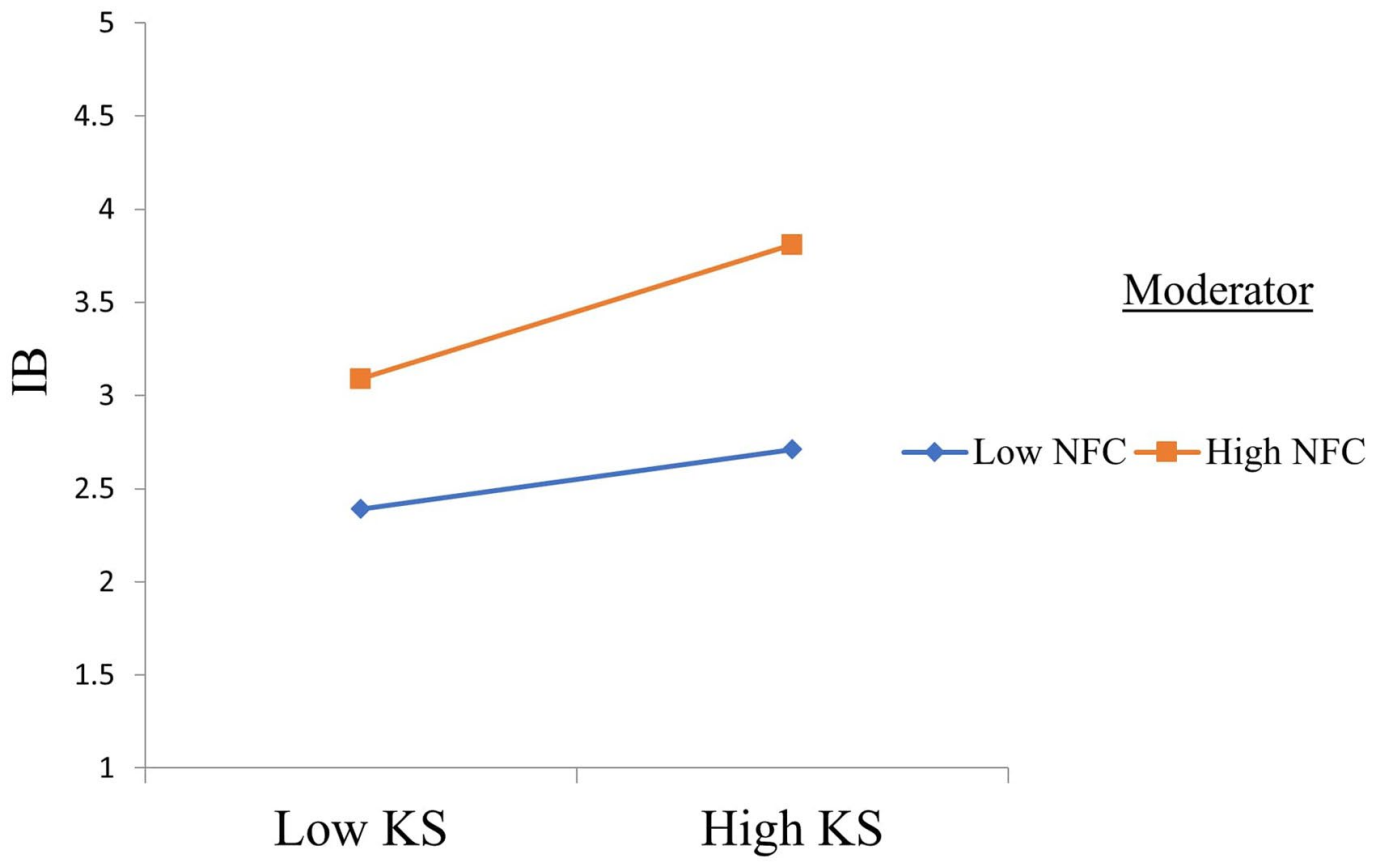
Interaction plot.

**Figure 3:**
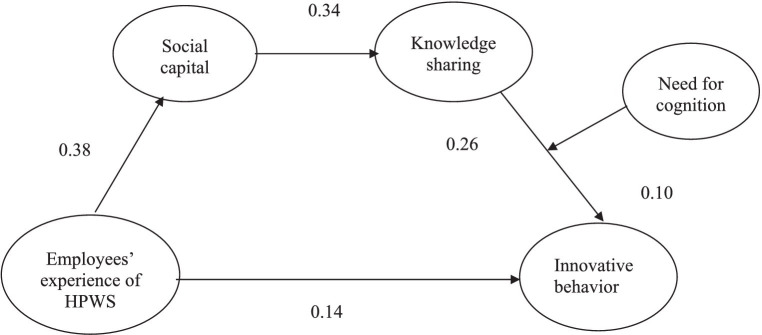
Results of hypotheses testing.

The primary focus of this study was to investigate the indirect relationships between employees’ experiences of HPWS and IB. Therefore, we examined our proposed mediation hypotheses. The results revealed that SC mediates the relationship between employees’ experience of HPWS and KS (β = 0.13, *p* < 0.01), as hypothesized in Hypothesis 4. Hypothesis 6 tested the mediating role of KS between SC and IB. The results confirmed the proposed mediation hypothesis (β = 0.09, *p* < 0.01). According to Hypothesis 7, SC and KS sequentially mediate the relationship between employees’ experience of HPWS and IB. The results confirm the proposed sequential mediation (β = 0.03, *p* < 0.05). H8 sought to ascertain the moderating role of NFC between KS and IB. The results revealed that the NFC moderated the relationship between KS and IB (β = 0.19, *p* < 0.05). The results indicate that, at a high NFC, KS was found to have a stronger impact on IB. The results of the structural model evaluation are presented in Table 4. The interaction plot in Figure 2 shows that with an increase in NFC, the positive relationship between KS and IB is strengthened. The results of the hypotheses testing are shown in Figure 3.

**Table 4:** Results of structural model evaluation.

Relationships	Path coefficients	*T* value	*p* value	Decision
Direct effects				
H1: HPWS➔IB	0.14	2.03	0.042	Supported
H2: HPWS➔SC	0.38	6.49	0.000	Supported
H3: SC➔KS	0.34	5.89	0.000	Supported
H5: KS➔IB	0.26	4.16	0.000	Supported
Indirect effects				
H4: HPWS➔SC➔KS	0.13	3.59	0.000	Supported
H6: SC➔KS➔IB	0.09	3.25	0.000	Supported
H7:HPWS➔SC➔KS➔IB	0.03	2.45	0.010	Supported
Total indirect effect				
Total effect				
HPWS➔IB	0.17	2.57	0.01	Supported
Moderation effect				
H8: NFC x KS	0.10	2.12	0.03	Supported

## 5 Discussion

This study investigates the relationship between employees’ experience of HPWS and IB. The results of the study validate our arguments based on the SET and ELM. We have used these theoretical perspectives to provide an in-depth understanding of how organizational, social, and cognitive factors interplay to influence IB. The results of the study highlight that social capital and knowledge sharing behavior sequentially mediate the relationship between HPWS and IB. Moreover, the results confirm the positive moderating influence of NFC on the relationship between KS and IB. The theoretical and practical implications of this study are presented in the following sections.

### 5.1 Theoretical contributions

This study makes several important contributions to the growing body of research on human resource systems and their influence on employee behaviors at the workplace. The SET suggests that employers establish long-term social relationships with employees through provision of benefits such as autonomy, rewards, and development opportunities. In exchange, employees repay the organization in the form of reciprocal behaviors aligned with the organizational goals (Kehoe and Collins, 2017; Alfes et al., 2021; Asante et al., 2023).

Consistent with the primary hypothesis and arguments based on the SET, we found that employees’ experience of HPWS promotes employees’ IB as a reciprocal social exchange behavior towards employer. The results support the notion of previous researchers that the perceived benefits of HPWS inculcate social exchange relationship between employer and employees (Bos-Nehles et al., 2017; Zhang et al., 2019; Zhu et al., 2022; Asante et al., 2023).

Furthermore, this study delves into the relational consequences of HPWS. Based on the tenets of SET, there is a growing debate that HRM practices contribute to the development of a social architecture that facilitates resource exchange and innovation (Oparaocha, 2016; Singh et al., 2021). This study contributes to this growing debate and highlights the role of interrelated human resource practices for engendering organizational innovation and competitive advantage.

Our study contributes to the research on HPWS and individual level innovation by highlighting social capital development and knowledge sharing as key relational links that underly the relationship. Existing studies have indicated that interrelated practices of HPWS have the potential to drive SC development in organizations (Donate et al., 2020; Wang et al., 2023). SC serves as a catalyst for innovation by facilitating exchange and flow of information ideas in the organization (Yen et al., 2015; Santos et al., 2023). Thus, SC can be considered as relevant mediator that underlys the relationship between HPWS and KS. Furthermore, HPWS encourages cooperation and relationship maintenance. In such contexts, employees may be inclined to share knowledge due to a shared belief that their contributions are valued (Ganguly et al., 2019; Donate et al., 2020). Consequently, KS fuels creative thinking, idea generation and implementation for value creation (Pian et al., 2019). Thus, the importance of KS as a mediator between SC and IB cannot be overlooked.

The results of the study support the argument that employees’ experiences of HPWS foster the development of social assets and exchange relationships (Evans and Davis, 2005; Jiang and Liu, 2015; Donate et al., 2020; Singh et al., 2021). The results also validate the arguments in the literature that SC serves as a bonding element that allows individuals to leverage useful knowledge for innovative value creation (Inkpen and Tsang, 2005; Hair et al., 2014; Ganguly et al., 2019; Al-Omoush et al., 2022). There is a paucity of research that delves deeply into the interplay of the underlying social and cognitive factors that facilitate individual innovation, particularly in PSF contexts. Previous studies have attempted to examine direct relationships or single mediation paths and neglected possible sequential paths that can provide an in-depth understanding of the relationships. Thus, this study clarifies the nature of this relationship between HPWS and IB by delving deeply into the underlying mechanisms through which HRM practices have an impact on IB. Despite the known benefits of SC in organizations, there is limited literature that explores its antecedents. Thus, another contribution of this study is that we have identified employee experience of HPWS as an enabling factor of SC development.

This study validates the arguments that KS interactions facilitate employees’ innovative behavior (Radaelli et al., 2014; Pian et al., 2019; Kmieciak, 2020). Additionally, this study is also a pioneering attempt to propose a moderating influence of individual NFC in the relationship between KS and IB. By using arguments based on ELM, we enrich the vast body of research on KS and innovation by identifying NFC as a boundary condition. NFC is a dispositional tendency to engage in in-depth information processing, cognitive elaboration, and critical thinking (Cacioppo and Petty, 1982). There is limited research that considers the influence of NFC on individual-level innovation (Wu et al., 2014; Pan et al., 2021). The results of the study posit that NFC drive IB by facilitating in-depth processing of knowledge shared during social interactions (Evans et al., 2003; Pan et al., 2021). This study suggests theoretical and practical relevance of NFC as a contextual factor that enhances employees ability to transform shared knowledge into innovative output. The current study underscores the pivotal significance of cognitive differences in amplifying the impact of KS on IB. In workplace contexts such as those which implement HPWS, individuals with high NFC can generate creative and innovative solutions through KS interactions (Wu et al., 2014). Our study suggests that individuals with high NFC are likely to contribute diverse ideas to shared knowledge pools.

Recent studies suggest that there are differences in managers’ and employees’ reports regarding the implementation of HRM practices in organizations. These studies suggest that an analysis of employees’ individual experiences of HRM practices on their behaviors can provide a better understanding as compared to the managerial perspective (Ali et al., 2022; Kim et al., 2023). In line with these arguments, this study uses employees’ reports to gauge their perceptions of HRM systems and resultant impact on their behaviors.

This study uses empirical evidence from PSFs in Pakistan, an emerging Asian country. PSFs play a vital role in the economic growth and development of emerging countries by promoting service exports. PSFs operate in a competitive business environment; therefore, employees’ IB is important to promote client satisfaction and achieve competitive advantage. Earlier research has documented the influence of HR practices on employee-related outcomes in various organizational contexts, such as manufacturing and high-technology firms (Park et al., 2017; Bos-Nehles and Veenendaal, 2019; Yao et al., 2023). Furthermore, there is limited evidence regarding the outcomes of HPWS in PSFs within developing country contexts. This study fills these research gaps by providing an understanding of different factors that can determine employees’ IB in PSF contexts. Although Pakistan is known as high power distance country, the results of this study dispel the widespread notion of bureaucratic culture and the lack of cooperation between employees in Pakistani organizations. Our study indicates that HPWS can provide benefits beyond cultural and societal boundaries.

### 5.2 Managerial implications

In addition to the above-mentioned theoretical contributions, this study also presents important practical implications. Our study highlights the relational dynamics of the HPWS, which can potentially lead to the effective utilization of knowledge utilization. This study advances the widespread understanding of managers that the HPWS not only contributes to human capital development but also influences social dynamics at workplaces. The findings illustrate that HPWS plays an important role in engendering social capital and knowledge sharing, which prompts employees to engage in IB. Thus, PSFs striving to compete in a dynamic business environment can implement HPWS to increase employees’ creative involvement. Moreover, this study also guides recruitment managers to assess potential job incumbents’ cognitive abilities, to promote innovation in PSFs. While this study focuses on PSFs in Pakistan, its findings can have broader implications for managers of PSFs in comparable countries in the region. This is because PSFs face similar challenges such as competitiveness and rapidly changing client demands.

### 5.3 Limitations and recommendations for future research

Despite its theoretical and practical implications, our study has some limitations that suggest avenues for future research. This study used employees’ self-reports to measure key study variables that may engender CMV. Future research can replicate our study using a combination of supervisor-rated and self-reported measures. Nevertheless, CMV was not a significant concern in this study as we employed statistical and procedural remedies to mitigate any potential biases. Moreover, this study used empirical data from PSFs in Pakistan. Data from a single geographical context may question the generalizability of the findings. Future research could employ cross-country comparisons for an in-depth understanding of the proposed relationships. Furthermore, future research can investigate additional mediating and moderating mechanisms that explain the relationship between employees experience of HPWS and IB.

## Data availability statement

The raw data supporting the conclusions of this article will be made available by the authors, without undue reservation.

## Ethics statement

The studies involving humans were approved by Dr. Mian Muhammad Atif, Chair, Research Ethics Committee, FAST School of Management, National University of Computer and Emerging Sciences, Lahore, Pakistan. The studies were conducted in accordance with the local legislation and institutional requirements. The participants provided their written informed consent to participate in this study.

## Author contributions

BA: Conceptualization, Formal analysis, Investigation, Methodology, Writing – original draft, Writing – review & editing. HH: Conceptualization, Methodology, Supervision, Writing – review & editing. AA: Conceptualization, Methodology, Writing – review & editing.
