# Supplementary material for: The impact of employees’ experience of high-performance work systems on innovative behavior in professional service firms
Source: Front Psychol. 2024 Jan 8;14:1324474. doi: 10.3389/fpsyg.2023.1324474 (PMC10800686; doi:10.3389/fpsyg.2023.1324474)
Supplement: Supplementary file 1 [file Data_Sheet_1.docx]

**Appendix 1**

**Questionnaire items**

**Employee experienced high performance work system** Adapted from Edgar et al., 2021

1. At work I have opportunity to participate in decision-making
2. I have a great deal of autonomy in the way I carry out my job.
3. I have good job security.
4. My organization gives fair performance-based rewards.
5. Training and development opportunities are provided.
6. My workplace sets very high standards and is very selective when recruiting staff.
7. Managers provide developmental feedback to improve performance.
8. I am provided with opportunities to express my ideas about how processes can be improved in this organization.
9. I believe my values fit well with those of my organization.
10. My organization values my ability to work well within a team environment.
11. My career goals are addressed through developmental feedback.
12. The ability to collectively solve problems is highly valued by my organization.

**Social capital** Adapted from Tsai 2014 and Leana and Pil (2006)

In my organization:

1. Employees demonstrate strong cohesiveness in workgroups.
2. Employees have frequent contact with coworkers.
3. Employees spend a lot of time interacting with each other.
4. Employees have close social relationships with each other.
5. There is a commonality of purpose among the employees.
6. Employees enthusiastically pursue collective goals and mission.
7. Employees are committed to the goals of the team/department/work unit.
8. Employees share similar ambitions and vision.
9. We can rely on co-workers and superiors, with whom we work.
10. Employees have confidence in one another.
11. There is high “team spirit” among employees.
12. Overall, the employees in my organization are trustworthy.

**Knowledge sharing** Adapted from Lin, 2007

1. When I have learned something new, I share my knowledge with my colleagues.
2. I share important work-related information with my colleagues.
3. I regularly tell my colleagues about my skills and tasks performed at work.
4. When I need certain knowledge, I ask my colleagues about it.
5. I like to be informed of what my colleagues know.
6. When I need to learn something, I ask my colleagues who have their technical knowhow, skills and abilities.
7. I think it is important that my colleagues know what information or knowledge I have.

**Need for cognition** Adapted from Lins de Holanda Coelho et al. (2020)

1. I would prefer complex rather than simple problems.
2. I like to handle situations that require a lot of thinking.
3. I find satisfaction in finding solutions to problems by thinking about them for a long time.
4. I prefer my life to be filled with interesting puzzles that I must solve.
5. I would prefer a task that is intellectual, challenging, and thoughtful to one that does not require much thought.
6. I really enjoy a task that involves coming up with new solutions to problems.

**Innovative behavior** Adapted from Jannsen (2000)

1. I often generate ideas to tackle complex issues at work.
2. I search out new working methods, techniques, or instruments for work.
3. I generate original and effective solutions for challenges faced by my team/work unit.
4. Where possible, I tend to mobilize support for innovative ideas given by my colleagues.
5. When I have an innovative idea, I talk to my manager(s) for approval.
6. I often encourage my colleagues to discuss new and innovative ideas for product/service/process improvements.
7. I adopt systematic ways of introducing innovative ideas into the work environment.
8. I evaluate the utility of innovative ideas before implementation.
9. I try my best to transform my ideas into useful applications/products/services/processes.

Note: Some items were removed during the analysis due to lower item loading values.
